# Use of Immunohistochemistry Techniques in Patients Exposed to Sulphur Mustard Gas

**DOI:** 10.4061/2011/659603

**Published:** 2011-07-06

**Authors:** Mostafa Ghanei, Marco Chilosi, Hassan Mohammad Hosseini Akbari, Rouzbeh Motiei-Langroudi, Ali Amini Harandi, Hassan Shamsaei, Moslem Bahadori, Henry D. Tazelaar

**Affiliations:** ^1^Research Center of Chemical Injuries, Baqiyatallah Medical Science University, Mollasadra Street, P.O. Box: 19945-546 , Tehran, Iran; ^2^Department of Pathology, University of Verona, Verona, Italy; ^3^Department of Pathology, Shaheed Beheshti University of Medical Sciences, Tehran, Iran; ^4^Division of Anatomic Pathology, Mayo Clinic Arizona, Scottsdale, AZ, USA

## Abstract

We performed a pathologic study with further using an immunohistochemical technique (using anti-p63 and anti-CK5) on tissues obtained by open lung biopsy from 18 patients with previous exposure to sulphur mustard (SM) as case group and 8 unexposed patients (control group). The most frequent pathologic diagnosis was constrictive bronchiolitis (44.4%), followed by respiratory (22.2%) and chronic cellular bronchiolitis (16.7%) in the case group, and hypersensitivity bronchiolitis (50%) in the control group. The pathologic diagnoses were significantly different in the case and control groups (*P* = 0.042). In slides stained by anti-p63 and anti-CK5, the percent of stained cells and the mean number of epithelial cells were lower in the case group in comparison to the control group. This difference was significant for the mean number of cells stained by anti-CK5 (*P* = 0.042). Furthermore, there was a significant correlation between pathologic diagnosis and total number of cells and mean number of cells stained with anti-p63 and anti-CK5 (P  value = 0.002, <0.001, 0.044). These results suggest that constrictive bronchiolitis may be the major pathologic consequence of exposure to SM. Moreover, decrease of p63 in respiratory tissues affected by SM may suggest the lack of regenerative capacity in these patients.

## 1. Introduction

Previous studies have reported that exposure to sulphur mustard (SM) can lead to the development of airway hyper-reactivity [1], chronic bronchitis, bronchiectasis, and lung fibrosis [2, 3], in chronic phase. However, recent studies have shown strong evidence that constrictive bronchiolitis may also be a main late complication in exposure to SM [2, 4–7]. Some studies have tried to illustrate the pathological features in persons exposed to SM. In a study using broncho-alveolar lavage (BAL), fibrosis, fibroblast proliferation, and increased collagen synthesis were observed in the patient respiratory parenchyma [4], consistent with the diagnosis of constrictive bronchiolitis [8, 9]. In another study obtaining tissue by BAL and transbronchial lung biopsy, evidence of organizing pneumonia or constrictive bronchiolitis with organizing pneumonia was observed [10]. Considering the value of pathology in the diagnosis however, immunostaining methods can confirm or further add to the data obtained from pathology, using antibodies against markers expressed in bronchial structures. Among the common used markers are cytokeratin (CK8) and surfactant (alveolar markers), CK5 and P63 (bronchial epithelial markers), CD34, CD31, and podoplanin (endothelial markers), and *α*-SMA (smooth muscle *α*-actin; fibroblast and myofibroblast marker) [11–13]. In our previous studies, we had shown that in SM-exposed patients, the pathology is centered in bronchioles. CK5 and P63 are bronchial epithelial markers which can help to detect remnants of bronchioles (vanished bronchioles, which are severely injured and distorted bronchioles) in cases in which conventional pathologic staining had failed to help. Immunohistochemistry may also provide additional information about the pathogenesis of SM. 

As no large comprehensive study has yet reported immunohistochemical features of people exposed to SM, we performed a collaborative international study in which we used antibodies against CK5 and P63 in tissues obtained by open lung biopsy from patients with previous exposure to SM.

## 2. Methods

### 2.1. Patient Population and Data Acquisition

Data were obtained from the medical records available at a major university hospital which provides tertiary medical care for patients exposed to chemical warfare agents during the Iran-Iraq war. The inclusion criteria were as follows: (1) patients who had experienced some level of exposure to SM and (2) patients who had undergone a surgical (open or thoracoscopic) lung biopsy, pulmonary function tests (PFTs), and chest high-resolution-computed tomography scan (HRCT). Patients were excluded if they had a history of significant occupational or other environmental exposures or a connective tissue disease. Smoking was not a reason for exclusion. Only two cases and none of controls were smokers, however. 18 patients finally met our inclusion criteria. Eight patients with the second inclusion criteria (as mentioned above) who had no history of sulphur mustard gas exposure were included in the study to serve as controls. These patients (in fact controls), were chosen among unexposed patients who presented with respiratory complaints and had undergone the above-mentioned evaluations. Cases and controls were selected in a consecutive manner.

All patients had signed an informed consent before participating in the study (including their consent to undergo a lung biopsy procedure) and all procedures were conducted in accordance with the principles of declaration of Helsinki, and the project design was approved by appropriate institutional ethics committee.

### 2.2. Surgical Procedure

Surgical procedures were performed under general anesthesia. If possible, biopsies were obtained from two different lobes of the lung at the interface between apparently normal and pathologic tissues. All specimens were fixed in formalin and embedded in paraffin according to standard laboratory procedures and sent to pathology lab immediately. Specimens were stained by hematoxylin-eosin, Masson's trichrome, and elastic tissue stains for pathologic review.

### 2.3. Immunohistochemical Procedure

Immunostaining was performed on formalin-fixed, paraffin-embedded sections using DAKO immunostaining methods (DAKO EnVision System, DAKO, A/S, Glostrup, Denmark), and all procedures were performed according to the manufacturer's protocols. Four-*μ*m-thick sections were deparaffinized, rehydrated, and treated with 3% hydrogen peroxide for 10 minutes to block endogenous peroxidase activity. For antigen retrieval, the sections were then heated in a microwave oven (750 W for 15 min with 0.01 M citric acid, pH 6.0). The sections were washed in phosphate-buffered saline (PBS) and incubated with the appropriate primary antibody overnight at room temperature. The antibodies used were as follows: anti-human cytokeratin 5/6 antibody (monoclonal mouse IgG1 antiserum, DAKO) for CK5; anti-human P63 antibody (monoclonal mouse antiserum, DAKO) for P63. For detection of primary antibodies, specimens were washed in PBS, followed by incubation with avidin-biotin-peroxidase complex according to the manufacturer's instructions. Hematoxylin was used as the nuclear counterstain. To evaluate the specificity of the antibody, known positive and negative tissues were used as controls. In order to grade the immunostaining, a standard power field was selected in all sections and then each section was divided into equal number of squares. The total number of bronchial epithelial cells, number of cells stained for p63 or CK5, and the percent of stained cells in each square were then calculated and used for further analysis. For anti-cytokeratin 5, diffuse, focal cytoplasmic, or membrane staining was scored as positive reactivity, and p63 expression was considered positive only if distinct nuclear staining was present.

### 2.4. Data Analysis and Review

All sections were initially studied in a blinded fashion without knowledge of clinical features by 6 pathologists from Iran (Research Center of Chemical Injuries, Baqiyatallah Medical Sciences University, Tehran and Department of pathology, Shaheed Beheshti University of Medical Sciences, Tehran), Italy (Department of Pathology, University of Verona), and USA (Department of Laboratory Medicine and Pathology, Mayo Clinic, Arizona). Afterwards, all cases were reviewed regarding complete clinical history, radiological findings, and collected immunohistochemical results. A final diagnosis was then determined for each patient. All data were analyzed with the commercially available software package SPSS (version 13.0).

## 3. Results

Between February 2004 and May 2005, 18 SM-exposed patients (all male, case group) and 8 unexposed patients (7 females and 1 male, control group) who met our inclusion criteria participated in this study. The mean ages of the cases and controls were 43.9 ± 9.6 (range 33–65) and 42.6 ± 10.8 (range 29–66), respectively, which was not significantly different (independent *t*-test, *P* = 0.85). The mean interval between exposure and involvement in the study for cases was 19.4 years (range 17–23). Two patients were smokers. All patients (100%) presented dyspnea and cough as their main complaint, while sputum production (60%), hemoptysis (46.7%), and chest pain (40%) were the other frequent complaints in patients. Obstructive lung pattern was seen in 11 (73.3%), mixed or restrictive pattern in 2 patients (13.3%), and 2 patients (13.3%) had normal pulmonary function test results. 

### 3.1. Pathological and Immunohistochemical Findings

Pathologic studies revealed that all cases had evidence of pathology centered on the small airways. As shown in Table 1, the most frequent diagnosis in the case group was constrictive bronchiolitis (8 patients; 44.4%) which was defined by partial luminal narrowing by the presence of plaque-like increases in circumferential or partial submucosal collagen. The next most common diagnoses were respiratory and chronic cellular bronchiolitis, which consisted of 4 (22.2%) and 3 (16.7%) patients, respectively. Other less frequent diagnoses were hypersensitivity bronchiolitis and nonspecific bronchiolitis. One specimen showed inadequate and equivocal findings which made a definite pathologic diagnosis impossible to make. The pathological diagnoses in the control group were hypersensitivity bronchiolitis (50%), bronchiolitis obliterans organizing pneumonia (BOOP) (12.5%), chronic cellular bronchiolitis (12.5%), constrictive bronchiolitis (12.5%), and neuroendocrine bronchiolitis (12.5%). Chi-square analysis showed that the pathologic diagnoses were significantly different in the case and control groups (*P* = 0.042) as there was a higher rate of constrictive bronchiolitis in the case group, in comparison to the control group.

Immunohistochemical staining for CK5 and p63 data is shown in Tables 2 and 3. As shown in Table 2, the percent of stained cells and the mean number of cells in slides stained by anti-p63 and anti-CK5 were lower, in the case group in comparison to the control group. Furthermore, independent *t*-test analysis showed that this difference was significant for the mean number of cells stained by anti-CK5 (*P* = 0.042). Also, the mean number of bronchioles and the mean number of intact bronchioles were lower and the mean number of injured bronchioles was higher in the case group in comparison to the control group; however, the differences were not statistically significant (independent *t*-test, *P* values =  0.47,  0.25, and  0.26, resp.). We further analyzed the correlation between each pathologic diagnosis and the Immunohistochemistry measures. One-way analysis of variance (ANOVA) showed that there was a significant effect of pathologic diagnosis on mean number of cells stained with anti-p63, mean number of cells stained with anti-CK5, and total number of cells (One-way ANOVA, *P* value  =  < 0.001,  0.044, and  0.002). The effect of pathologic diagnosis on percent of cells stained by anti-p63 or anti-CK5, mean number of vanished, intact, injured and total bronchioles was not significant (One-way ANOVA, *P* values  =  0.26,  0.40,  0.24,  0.95,  0.59, and  0.97, resp.). The mean number of cells and the percent of cells stained by anti-p63 and anti-CK5 are shown in Table 3.

## 4. Discussion

**Figure 1:**
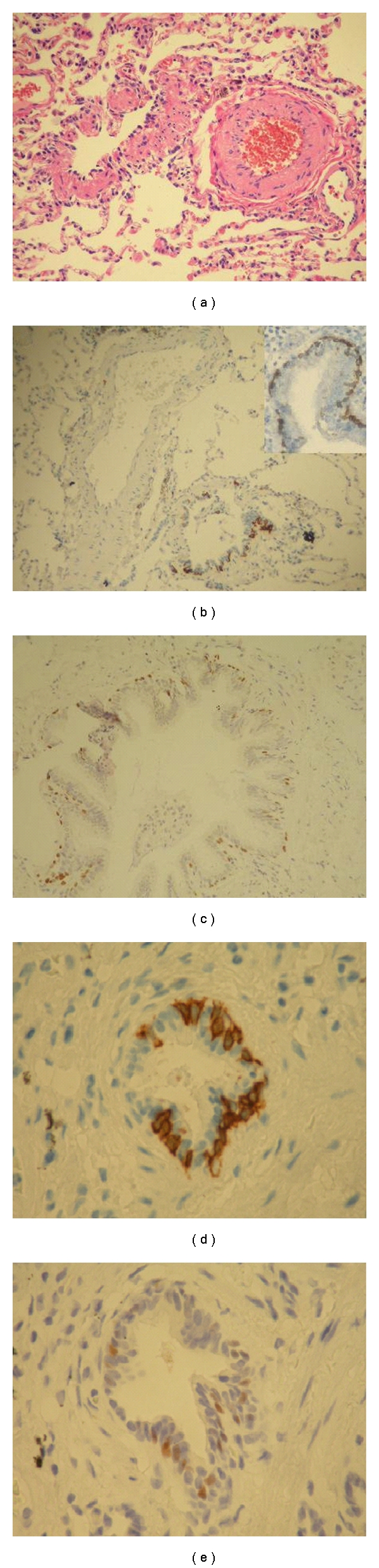
Pathologic evidence of constrictive bronchiolitis in a mustard-gas-exposed patients, stained with H & E (a), anti-CK5 (b), and anti P63 (c), respectively. (d) and (e) show anti-CK5 and anti-P63 staining in an unexposed patient (control) diagnosed as hypersensitivity pneumonitis.

SM is reported to cause ongoing injury that may not manifest itself for many years; among them, the most common late complications of exposure are respiratory complications. Previous studies have mostly reported chronic bronchitis, asthmatoid bronchitis, lung fibrosis, bronchiectasis, and bronchial stenosis as the main late complications of exposure [3]. However, none of these studies have assessed the respiratory effects of SM exposure pathologically, and most of these studies are based on clinical, spirometric, and/or radiological findings, which have their own pitfalls. Therefore, some recent more comprehensive and multicenter studies, adding pathology findings to their current radiological data, have tried to further increase the reliability of their findings. This way, a number of studies have shown some evidence that constrictive bronchiolitis may be the main late complication in patients exposed to SM [2, 4, 7, 10]. In the current study, this was further confirmed, as we observed more patients pathologically diagnosed as constrictive bronchiolitis in the group previously exposed to sulphur mustard (Figure 1).

**Figure 2:**
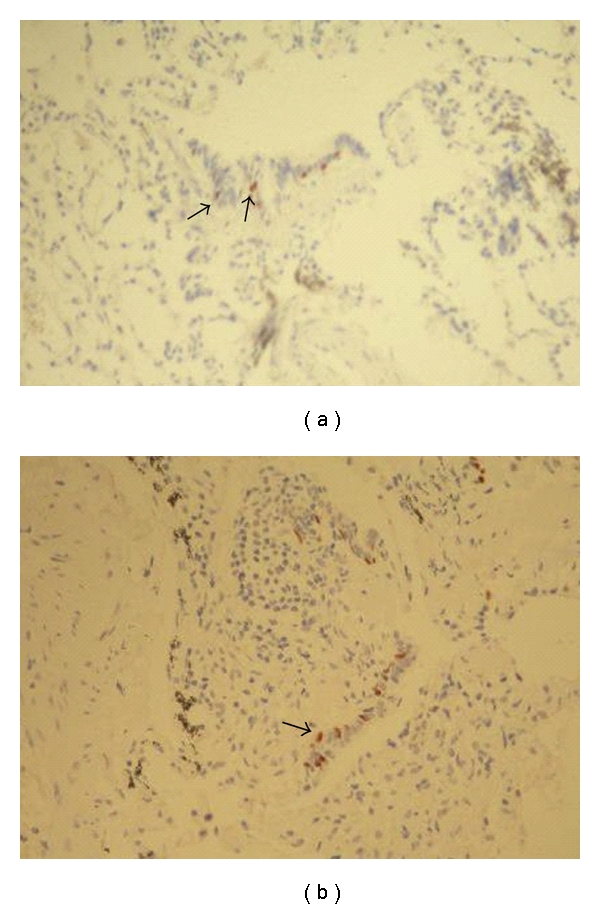
Evidence of vanished bronchioles in 2 mustard-gas-exposed patients. Scant staining, with anti-P63 (arrows), distinguishes the remnants of injured bronchioles from nonbronchiolar tissue.

Constrictive bronchiolitis is a relatively rare disease which occurs mostly in patients with a history of previous childhood infections, toxic fume inhalation (such as diacetyl [1] and SM [3, 7, 14, 15], graft-versus-host disease and chronic rejection following bone marrow, lung or heart-lung transplantation [16, 17], and other clinical settings. Pathologically, it is characterized by a distinctive pattern of chronic peribronchiolar inflammation, infiltration, and fibrosis which surrounds rather than fills the lumen, ultimately resulting in airway obstacle, cicatrisation of the bronchiolar lumen, and airway loss [4, 18]. Some other types of bronchiolitis are in the differential diagnosis of constrictive bronchiolitis, including respiratory bronchiolitis and cellular bronchiolitis. Respiratory bronchiolitis is defined by the presence of pigmented macrophages within the lumen of distal airways, not associated with significant inflammation, fibroblast activity, or collagen deposition. In cellular bronchiolitis, the bronchioles show an increased number of acute (neutrophils) or chronic (lymphocytes, plasma cells, macrophages, or tan-brown histiocytes) inflammatory cells (luminal, mural, or both), accompanied by necrosis of epithelial and inflammatory cells, submucosal edema or necrosis, neutrophil microabscesses, and germinal center hyperplasia (follicular bronchiolitis). However, advanced cases of constrictive bronchiolitis may be especially difficult to differentiate because of lack of active inflammation and disappearance of bronchioles (or *vanished bronchioles*). In such cases, other staining methods such as elastic and immunostaining may be useful in identification of affected structures, by defining specific markers [18]. Regarding this, we observed evidence of vanished bronchioles in 4 patients, which helped us make a definite pathologic diagnosis (Figure 2). 

A number of immunohistochemical markers have been suggested as useful markers for epithelial cells. Among these are cytokeratin (CK5) and p63. CK5, an intermediate molecular weight keratin, is normally present in basal cells of complex normal epithelia, such as respiratory, squamous, myoepithelial, and certain ductal epithelia [13, 19]. CK5 serves as the cytoplasmic marker of bronchial basal epithelial cells. P63 is a p53 homologue which, in normal tissues, is present in the basal and suprabasal cell population of stratified epithelia such as bronchi and lost in more superficial, terminally differentiated cell layers [11, 20]. Our immunohistochemistry results further added some evidence about SM-mediated injury. Exposure to sulphur mustard significantly decreases the total number of epithelial cells. It also decreases the number of cells stained for CK5 and p63 markers, and total and intact bronchioles. However, the latter decreases were not significant, may be because of our limited number of patients and also because our control group consisted of unexposed patients and not normal people. It is suggested that SM undergoes intramolecular cyclization to form intermediates, which, in turn, react with and alkylate cell constituents, mainly DNA, and also RNA, proteins, and lipid membranes [21, 22]. These reactions may result in physiological, metabolic, and genetic failure of cellular functions. Moreover, we showed that the pathologic diagnosis significantly correlated with these measures. In patients who were diagnosed as constrictive bronchiolitis, the number of epithelial cells and cells stained for CK5 and p63 were lower, in comparison to the other diagnoses. P63 has been shown to play a critical role in development of stratified epithelia by maintaining basal cell regenerative capacity [23, 24]. Some isoforms of the p63 gene counteract the apoptotic and cell cycle inhibitory functions of p53 after DNA damage, and this property is likely to be central in the cell renewal strategy of stratified epithelial tissues [25]. 

In summary, our results suggest that constrictive bronchiolitis may be the major pathologic consequence of exposure to SM. Moreover, decrease of p63 in respiratory tissues affected by SM may suggest the lack of regenerative capacity in these patients. Further studies are needed to elucidate the detailed mechanisms of SM action.

## Figures and Tables

**Table 1:** Pathologic diagnosis of cases and controls.

Case group		Control group	
Diagnosis	Number of cases (%)	Diagnosis	Number of cases (%)
Constrictive bronchiolitis	8 (44.44)	Hypersensitivity bronchiolitis	4 (50)
Respiratory bronchiolitis	4 (22.22)	Bronchiolitis obliterans organizing pneumonia (BOOP)	1 (12.5)
Chronic cellular bronchiolitis	3 (16.67)	Chronic cellular bronchiolitis	1 (12.5)
Hypersensitivity bronchiolitis	1 (5.56)	Constrictive bronchiolitis	1 (12.5)
Nonspecific bronchiolitis	1 (5.56)	Neuroendocrine bronchiolitis	1 (12.5)
Indefinite	1 (5.56)		

**Table 2:** IHC findings in control and case groups. The number of cells represents the number of cells (whether stained or not) in each square, as described in the methods. Each square in all slides was approximately equal to 0.7980 mm^2^. The number of bronchioles is represented in a slide observed in a low-power field view. *P < 0.05 compared to the relevant control group.

	P63 staining		CK5 staining		Number of bronchioles		
	Mean number of cells (± S D)	Percent of stained cells (± S D)	Mean number of cells (± S D)	Percent of stained cells (± S D)	Total	Intact	Injured
Control	13.89 ± 3.33	50.16 ± 3.15	16.44 ± 2.44	50.94 ± 30.36	3.0	2.67	0.33
Case	12.11 ± 5.21	42.11 ± 19.32	10.99 ± 3.99*	37.20 ± 22.50	2.44	1.83	0.61
*P* value	0.43	0.16	0.042	0.34	0.47	0.25	0.26

**Table 3:** The IHC data, regarding each pathologic diagnosis. The number of cells represents the number of cells (whether stained or not) in each square, as described in the methods. Each square in all slides was approximately equal to 0.7980 mm^2^. The number of bronchioles is represented in a slide observed in a low-power field view.

	P63 staining		CK5 staining		Number of bronchioles (intact + injured)	Percent of patients with vanished bronchioles
	Mean number of cells (± S D)	Percent of stained cells (± S D)	Mean number of cells (± S D)	Percent of stained cells (± S D)		
Constrictive bronchiolitis	9.62 ± 3.19	39.12 ± 15.88	10.27 ± 2.65	26.40 ± 13.80	2.63 (2.00 + 0.63)	75.0
Chronic cellular bronchiolitis	18.24 ± 2.00	47.38 ± 24.00	13.99 ± 2.56	57.72 ± 7.30	2.25 (1.75 + 0.50)	33.3
Hypersensitivity pneumonitis	13.08 ± 3.26	47.22 ± 6.15	12.08 ± 3.25	55.74 ± 31.30	2.80 (2.60 + 0.20)	25.0
Respiratory bronchiolitis	13.71 ± 3.35	50.17 ± 38.24	11.33 ± 6.85	39.79 ± 33.67	2.25 (1.75 + 0.50)	0
Neuroendocrine bronchiolitis	18.57	26.67	19.14	15.85	3.00 (2.00 + 1.00)	100.0
Nonspecific bronchiolitis	12.15	40.73	10.29	39.92	2.00 (1.00 + 1.00)	100.0
